# Supplementary material for: In situ transcriptome characteristics are lost following culture adaptation of adult cardiac stem cells
Source: Sci Rep. 2018 Aug 13;8:12060. doi: 10.1038/s41598-018-30551-1 (PMC6089936; doi:10.1038/s41598-018-30551-1)
Supplement: Supplementary file 1 — Supplementary Information [file 41598_2018_30551_MOESM1_ESM.docx]

**Supplementary Information**

***In situ* transcriptome characteristics are lost following culture adaptation of adult cardiac stem cells**

Taeyong Kim^1^, Oscar H. Echeagaray^1^, Bingyan J Wang^1^, Alexandria Casillas^1^, Kathleen M. Broughton^1^, Bong-Hyun Kim^2^, and Mark A Sussman^1,^*

**Detailed Experimental Procedures**

**Isolation of c-Kit^+^ / Lin^-^ CPC populations**

Adult c-Kit+ CPCs were isolated and expanded as previously described^1^. Twelve-week-old FVB female mice were heparinized and anesthetized by ketamine-xylazine solution. Hearts were removed from the chest cavity and perfused on a Langendorff system at 1mL/min flow rate at 37ºC with perfusion buffer (NaCl, KCl, KH2PO4, Na2HPO4, MgSO4•7H2O, NaHCO3, KHCO3 HEPES, Taurine, Glucose, and BDM) for blood removal, and tissue was subsequently digested for 10-15 minutes with Liberase DH digestion buffer (Roche 05401089001, 5 mg/mL in perfusion buffer). Heart was collected in STOP buffer (10% Bovine calf serum, 12.5 µM CaCl2), minced into 1 mm^3^ and dissociated through pipetting. The non-myocyte population was separated through serial cell straining (100 µm, 40 µm, and 30 µm pore size), followed by centrifugation at ~300 rcf for 10 minutes. c-Kit^+^ / Lin^-^ CPCs were obtained by immunomagnetic sorting with Lineage depletion kit and CD117-conjugated Microbeads (Miltenyi Biotech 130-048-102). Fresh isolated CPCs were separated for immediate single-cell RNA-Seq analysis.

All experiments involving mice and use of vertebrate animals were carried out according to Institutional Review Boards (IRB) policy and approved by the Institutional Animal Care and Use Committee (IACUC) at San Diego State University.

**CPC *in vitro* Culturing**

Upon isolation, CPCs from two individual hearts were pooled and plated on a pre-coated (0.1% porcine gelatin, 30 min at 37ºC) 35 mm tissue culture dish in growth medium (Dulbecco's modified Eagle's medium, 10% embryonic stem cells FBS, 1% insulin-transferrin-selenium, leukemia inhibitory factor [10 ng/mL], basic fibroblast growth factor [10 ng/mL], epidermal growth factor [20 ng/mL], L-glutamine [0.07 mg/mL], 1% penicillin-streptomycin-glutamine, 0.1% gentamicin, 2% B27 supplement, 1% N2 supplement). After five-seven days expansion, all adherent cells were passaged using 0.25% Trypsin/EDTA into a gelatin pre-coated 60 mm TC dish. For further expansion, adherent cells were expanded in growth medium when 40% confluency was reached and plated at approximately 200,000 cells on gelatin pre-coated 100 mm TC dish. All culturing conditions were kept in humidified incubator at 37ºC with 5% CO_2_ under normoxic condition (ambient air). Cells at passage 5 were used for scRNA-seq.

**Single-cell RNA-seq**

**10X Genomics platform**: Freshly isolated and cultured CPCs were washed with PBS, centrifuged and resuspended in 0.04% non-acetylated bovine serum albumin to an approximate cell concentration of 200 cells/μl. Cell suspensions were loaded on a Chromium™ Controller (10X Genomics) to generate single-cell Gel Bead-In-EMulsions (GEMs). Single-cell RNA-Seq libraries were prepared using Chromium™ Single Cell 3’ Library & Gel Bead Kit v2 (10X Genomics). GEM-reverse transcription (RT) was performed in a C1000 Touch Thermal cycler with TempAssure PCR 8-tube strip (USA Scientific): 55 °C for 2 h, 85 °C for 5 min; held at 4 °C. After RT, GEMs were broken, and the single-strand cDNA was cleaned up with DynaBeads MyOne Silane Beads (Thermo Fisher Scientific) and SPRIselect Reagent Kit (0.6X SPRI; Beckman Coulter). cDNA was amplified using the C1000 Touch Thermal cycler with TempAssure PCR 8-tube strip: 98 °C for 3 min; cycled 14X: 98 °C for 15 sec, 67 °C for 20 sec, and 72 °C for 1 min; 72 °C for 1 min; held at 4 °C. Amplified cDNA product was cleaned up with the SPRIselect Reagent Kit (0.6X SPRI). Indexed sequencing libraries were constructed using the reagents in the Chromium™ Single Cell 3’ Library & Gel Bead Kit v2, following these steps: (1) end repair and A-tailing; (2) adapter ligation; (3) post-ligation cleanup with SPRIselect; (4) sample index PCR and cleanup. For quality control of libraries, each library was tested with Bioanalyzer (average library size: 450-490 bp). The sequencing libraries were quantified by quantitative PCR (KAPA Biosystems Library Quantification Kit for Illumina platforms P/N KK4824) and Qubit 3.0 with dsDNA HS Assay Kit (Thermo Fisher Scientific). Sequencing libraries were loaded at 2 pM on an Illumina HiSeq2500 with 2X75 paired-end kits using the following read length: 98 bp Read1, 8 bp i7 Index, and 26 bp Read2.

**Smart-Seq2 platform:** Cultured CPCs were harvested by trypsinization and centrifuged for 5 min at 300 rcf, 4°C. CPCs were resuspended to in PBS and single cells were captured under stereomicroscope by mouth pipetting with a ~0.2 mm diameter flame-pulled glass Pasteur pipet attached to aspirator tube (Sigma-Aldrich, A5177). Selected cells were rinsed >7 times in PBS and dispensed into Eppendorf tube containing 10 μL cell lysis buffer provided by Smart-Seq v4 kit. cDNA was synthesized following manufacturer’s protocol (Smart-Seq v4 ultra low amount cDNA kit Clontech, 634888). cDNA was validated on Agilent Bioanalyzer using High sensitivity DNA kit (Agilent; 5067-4626). Illumina sequencing libraries were then constructed using the Nextera XT DNA Sample Preparation kit (Illumina, FC-131-1024). Quality control of constructed libraries was performed on Agilent Bioanalyzer, and library quantification done by qPCR with KAPA library quantification kit (Kapa Biosystems, KR0405) and Qubit™ with dsDNA HS Assay Kit (ThermoFisher Scientific, Q32851). The pooled libraries were sequenced as paired-end 75x75 base reads on a NextSeq 500 with mid-output kit.

**Data Analysis**

**10X Genomics platform**: The raw data was processed with the Cell Ranger pipeline (10X Genomics; version 2.0). Sequencing reads were aligned to the mouse genome mm10. 1,615 and 850 cells were recovered from freshly isolated and cultured CPC samples, respectively. Cells with fewer than 1,000 genes or more than 10% of mitochondrial gene UMI count were filtered out and genes detected fewer than in three cells were filtered out^2^. Altogether, 2,383 cells and 15,786 genes were kept for downstream analysis using Seurat R Package (v2.3.0). Approximately 2,000 variable genes were selected based on their expression and dispersion (expression cutoff = 0.0125, x.high.cutoff =6, and dispersion cutoff = 0.5). The first 15 principal components were used for the t-SNE projection^3^ and unsupervised clustering^2^. Gene expression pathway analysis was performed using clusterProfiler.^4^

**Smart-Seq2 platform:** Smart-Seq2 scRNA-seq datasets were obtained from public databases (Supplementary table) with exception of the CPC dataset generated for this study. Downloaded datasets are from three different databases: Gene Expression Omnibus (GEO), ArrayExpress or Genomic Experiment Data Repository and Core LIMS database (GNomEx database). Sequencing reads were mapped to UCSC mouse genome mm10 using STAR v2.5.2b^5^ with default parameters and only uniquely mapped reads were kept. Read counts table was used as an input for generating Seurat object. Cells with fewer than 1,000 genes or more than 10% of mitochondrial gene count were filtered out and genes detected fewer than in three cells were filtered out. To exclude effects of sequencing depth variation, scRNA-seq raw data were downsampled to 500,000 reads per cell by random selection of raw read from fastq files and re-analyzed. 18,698 genes and 1,126 cells were kept for further analysis. Clustering analysis and downstream analysis were performed as outlined in the 10X Genomics platform section.

**Statistics**

Significant differences in the number of genes detected between fresh and cultured datasets from both platforms were analyzed with Wilcoxon matched-pairs rank sum test, with statistical significance accepted when p < 0.05.

**References**

1. Konstandin, M. H. *et al.* Fibronectin is essential for reparative cardiac progenitor cell response after myocardial infarction. *Circ. Res.* **113**, 115-125 (2013).

2. Macosko, E. Z. *et al.* Highly Parallel Genome-wide Expression Profiling of Individual Cells Using Nanoliter Droplets. *Cell* **161**, 1202-1214 (2015).

3. van der Maaten, L. & Hinton, G. Visualizing Data using t-SNE. *J. Mach. Learn. Res.* **9**, 2579-2605 (2008).

4. Yu, G., Wang, L. G., Han, Y. & He, Q. Y. clusterProfiler: an R package for comparing biological themes among gene clusters. *OMICS* **16**, 284-287 (2012).

5. Dobin, A. *et al.* STAR: ultrafast universal RNA-seq aligner. *Bioinformatics* **29**, 15-21 (2013).

**Supplementary Figures**


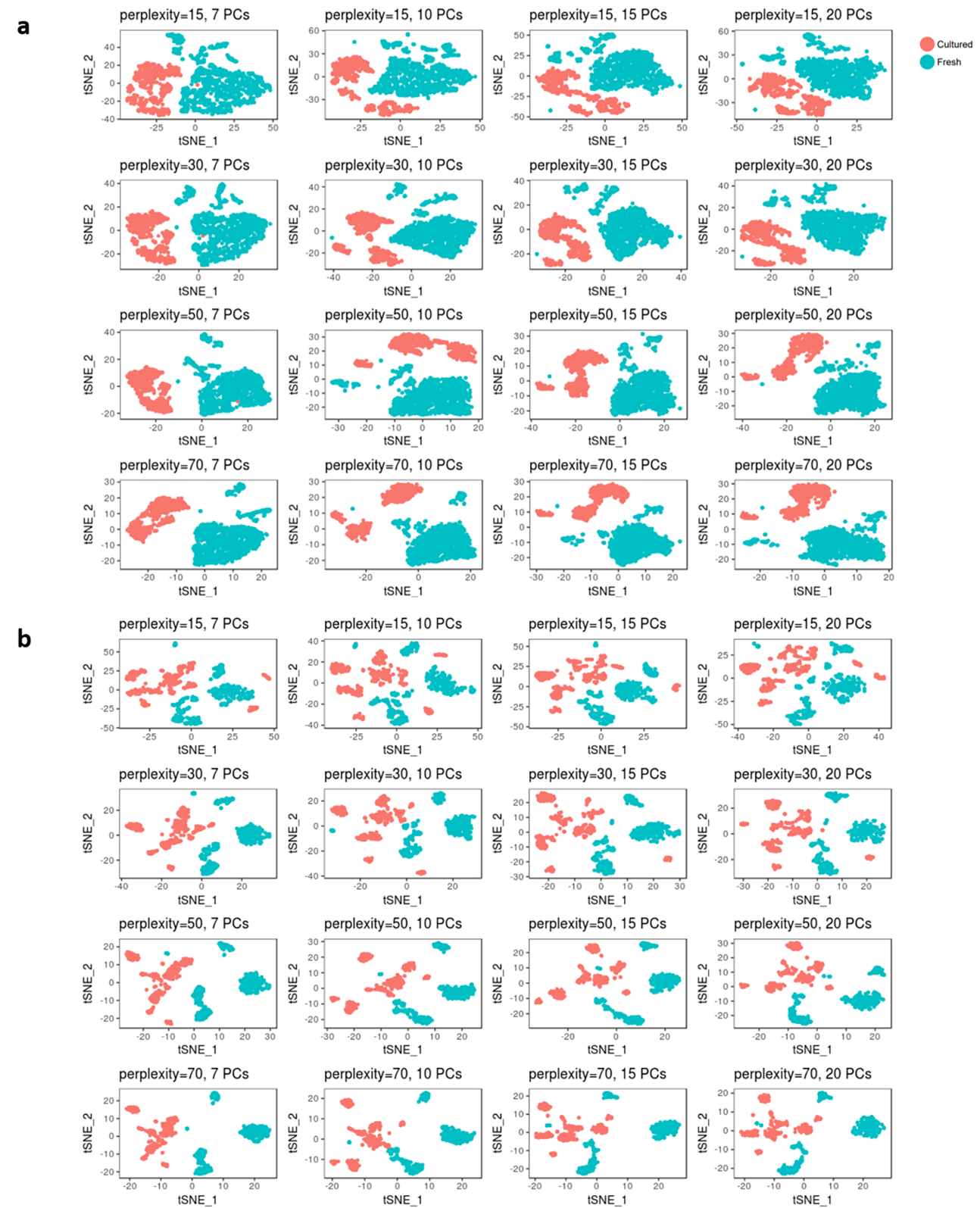


**Figure S1. Freshly isolated cells clustered separated from cultured cell clusters regardless of parameter setting in 10X genomics data (a) or Smart-Seq2 data (b).** Robustness test with 4 different perplexity numbers (15, 30, 50, and 70) and 4 different number of PCs (7, 10, 15, and 20) shown in t-SNE plots.


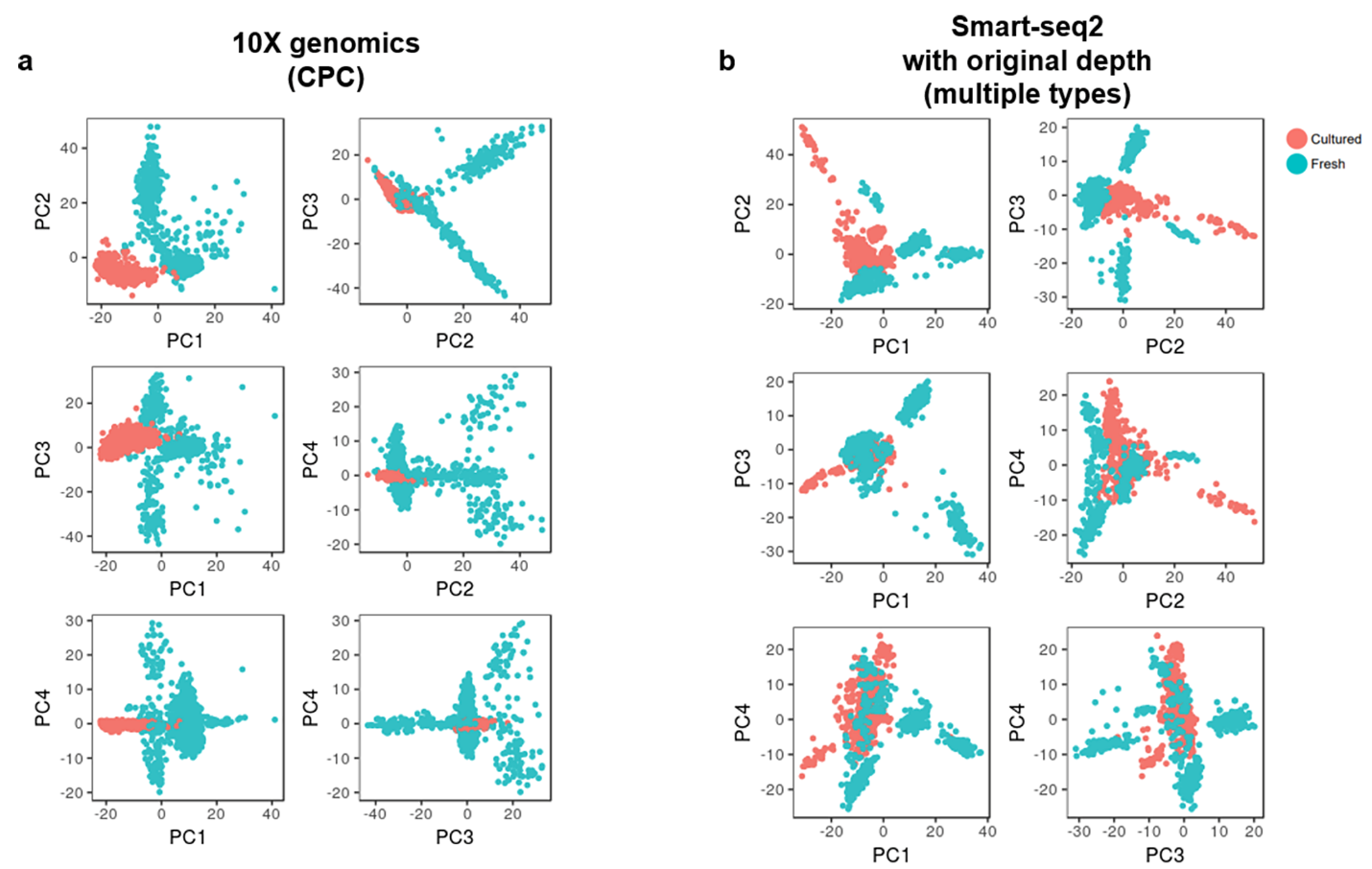


**Figure S2. First four principle components depict relatively tight clustering of cultured cells away from fresh cells in 10X genomics data (a) or Smart-Seq2 data (b).** Combination of the four PCs shown in PCA plots.


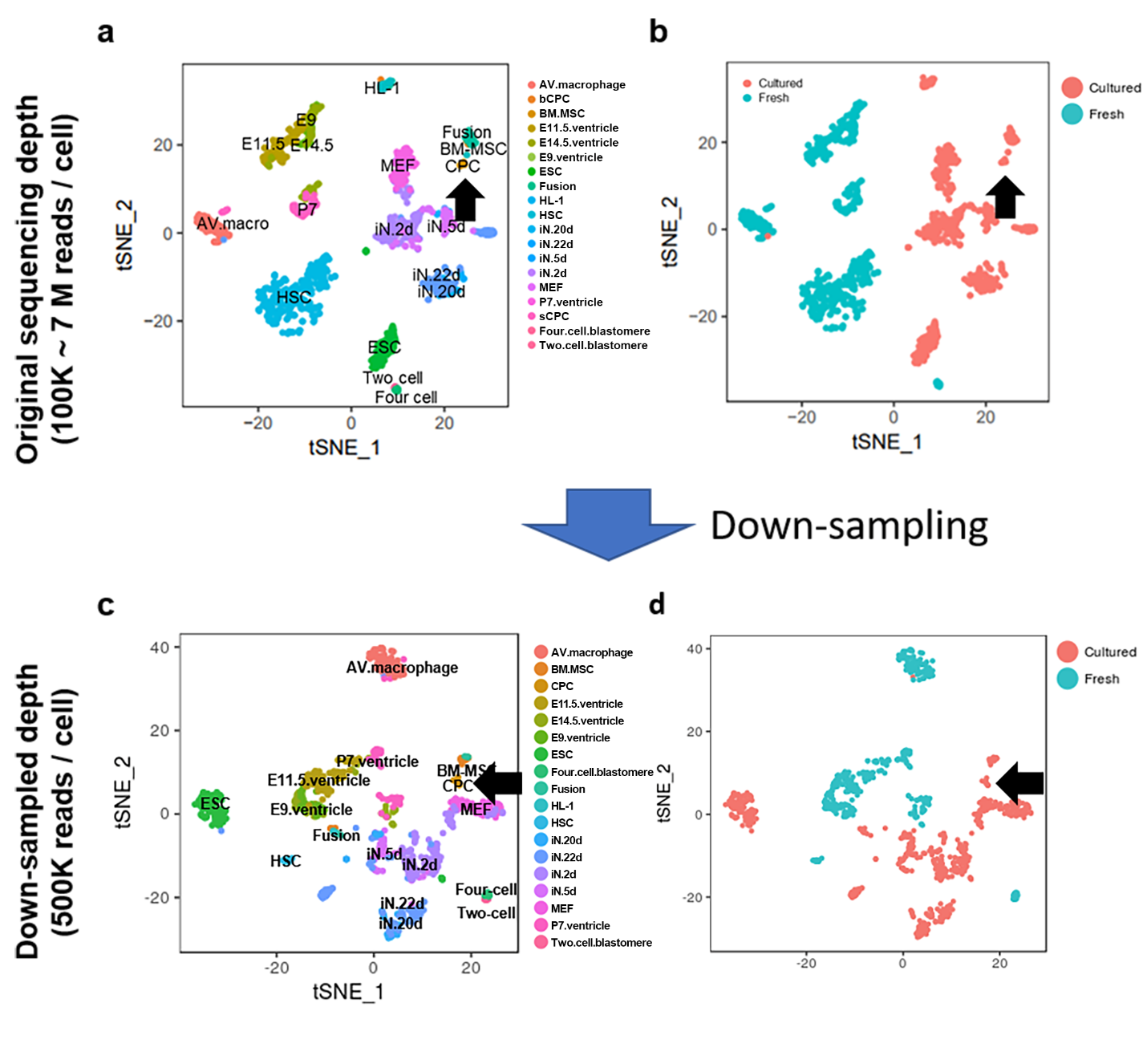


**Figure S3. Cultured cells are clearly different from fresh cells even after downsampling.** (a-d) t-SNE plots show scRNA-seq data generated by Smart-Seq2 before and after downsampling. Single cells are labeled by cell types (a and c) or environments (b and d). Arrow indicates CPCs prepared and sequenced in this study relative to single-cell datasets downloaded from public databases.

**
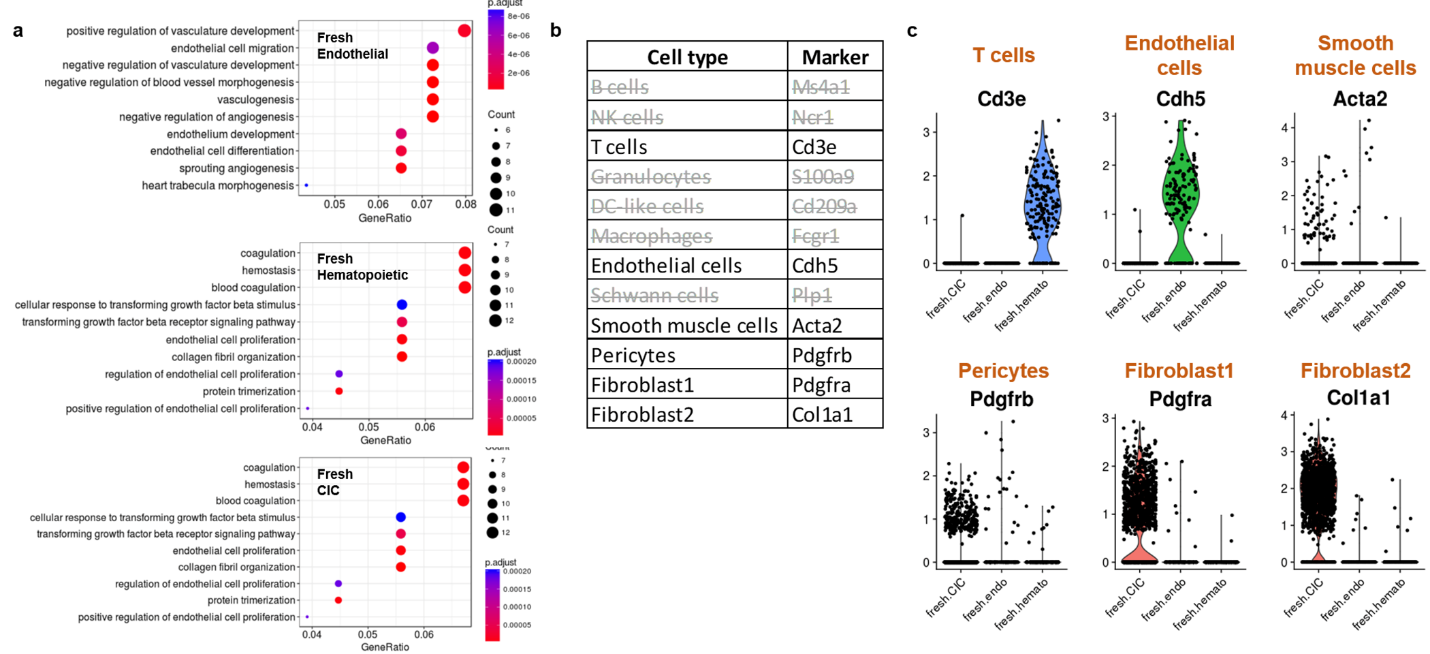
**

**Figure S4. Identification of fresh cell clusters**

(a) Dotplots representing gene set enrichment analysis with marker genes of three fresh cell clusters. (b) List of marker genes of cell types adopted from Skelly et al [ref. 11]. Markers with strikethrough were not detected in our scRNA-seq dataset, indicating likely depletion due to Lin^-^cKit^+^ sorting in our protocol. (c) Three fresh cell clusters are identified based on markers derived from Skelly et al [ref. 11]. ‘fresh.hemato’ cluster is T cell, ‘fresh.endo’ cluster is endothelial cell, and ‘fresh.CIC’ cluster has smooth muscle cells, pericytes, and two different fibroblasts.

**
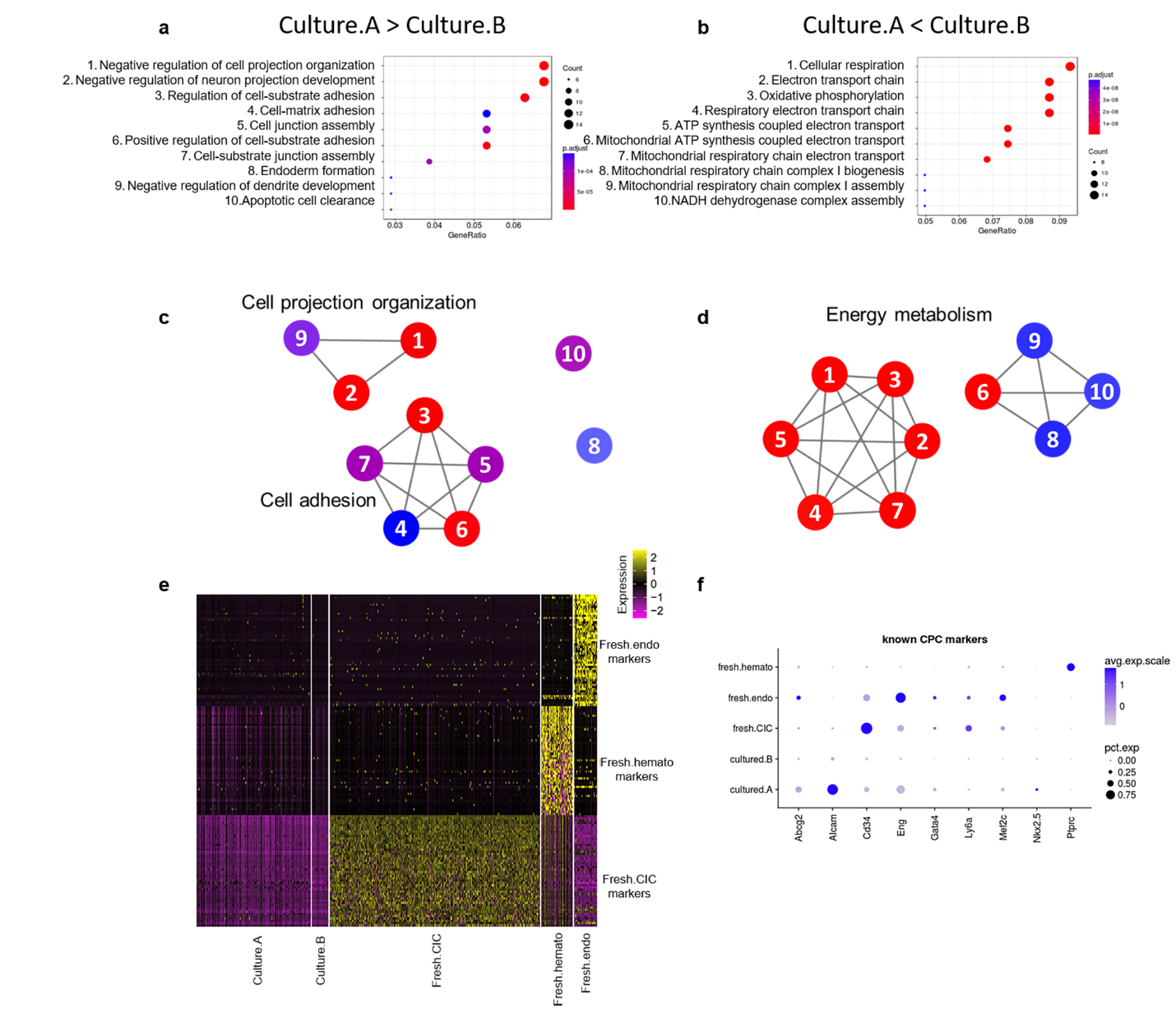
**

**Figure S5. Identification of cultured cell clusters.**

(a and b) Dotplots represent top 10 GO terms up-regulated in cultured cluster A revealed by gene set enrichment analysis from 10X Genomics (a) or Smart-Seq2 data analysis (b). Red indicates higher enrichment, blue indicates lower enrichment. The sizes of the dots represent the number of DEGs belong to each GO term. (c and d) Correlation network of gene sets up-regulated in cultured cells from 10X genomics (c) or Smart-Seq2 data analysis (d). Closely related terms are linked to each other. Cell proliferation-related terms are labeled with the name of GO terms. (e) Heatmap representing expression of top 50 marker genes of three fresh clusters. (f) Dotplot representing expression level of known CPC markers in CPC clusters (CPC markers were adopted from ref. 29 Smith et al.). Individual dots are sized to reflect the proportion of cells of each type expressing the marker gene and colored to reflect the mean expression of each marker gene across all cells, as indicated in the key.

**Supplementary Table. single-cell RNA-sequencing datasets generated by using Smart-Seq2 technology were downloaded from database.**

* Not all samples from the original paper were available in GNomEx database.

| **Dataset ID** | **Cell type** | **Fresh/cultured** | **Reference (PMID)** |
| --- | --- | --- | --- |
| GSE69926 | BM-MSC | Cultured | 26997336 |
|  | HL-1 |  |  |
|  | Fusion cell of BM-MSC and HL-1 |  |  |
| E-MTAB-2600 | ESC | Cultured | 26887813 |
| GSE67310 | MEF | Cultured | 27281220 |
|  | Induced neuron |  |  |
| GSE68981 | HSC | Fresh | 28003475 |
| GNomEx (272R, 274R, 275-292R, 439R, and 440R) * | Embryonic/postnatal cardiomyocyte | Fresh | 27840107 |
|  | Embryonic/postnatal fibroblast |  |  |
|  | Embryonic/postnatal endothelial cell |  |  |
| GSE86310 | atrioventricular node macrophage | Fresh | 28431249 |
| GSE57249 | 2-cell mouse embryos | Fresh | 25096407 |
|  | 4-cell mouse embryos |  |  |
